# Emotion in Nonverbal Communication: Comparing Animal and Human Vocalizations and Human Text Messages

**DOI:** 10.1177/17540739241303505

**Published:** 2025-01-15

**Authors:** T. Gruber, E. F. Briefer, A. Grütter, A. Xanthos, D. Grandjean, M. B. Manser, S. Frühholz

**Affiliations:** Swiss Center for Affective Sciences and Faculty of Psychology and Educational Sciences, University of Geneva, Switzerland; Behavioural Ecology Group, Section for Ecology & Evolution, Department of Biology, University of Copenhagen, Copenhagen Ø, Denmark; Department of Language and Information Sciences, Faculty of Arts, University of Lausanne, Lausanne, Switzerland; English Seminar, University of Zurich, Zurich, Switzerland; Department of Language and Information Sciences, Faculty of Arts, University of Lausanne, Lausanne, Switzerland; Swiss Center for Affective Sciences and Faculty of Psychology and Educational Sciences, University of Geneva, Switzerland; Department of Evolutionary Biology and Environmental Studies, University of Zurich, Zurich, Switzerland; Center for the Interdisciplinary Study of Language Evolution, University of Zurich, Zurich, Switzerland; Cognitive and Affective Neuroscience Unit, University of Zurich, Zurich, Switzerland; Department of Psychology, University of Oslo, Oslo, Norway

**Keywords:** emotional communication, animal vocalizations, prosody, evolution of communication

## Abstract

Humans and other animals communicate a large quantity of information vocally through nonverbal means. Here, we review the domains of animal vocalizations, human nonverbal vocal communication and computer-mediated communication (CMC), under the common thread of emotion, which, we suggest, connects them as a dimension of all these types of communication. After reviewing the use of emotions across domains, we focus on two concepts that have often been opposed to emotion in the animal versus human communication literature: control and meaning. Non-human vocal communication is commonly described as emotional, preventing either control or meaning; in contrast, the emotional dimension of human nonverbal signals does not prevent them from being perceived as both intentionally produced and meaningful. Amongst others, we disagree with this position, highlighting here that emotions should be integrated across species and modalities such as the written modality. We conclude by delineating ways in which each of these domains can meaningfully influence each other, and debates in their respective fields, and more generally the debate on the evolution of communication.

## Introduction

Over the last few decades, the topics of communication and emotion have increasingly been connected in a diversity of domains of research, such as human communication, non-human animal (from henceforth, animal) communication, and more recently in computer-mediated communication (CMC). This has been triggered either by basic interests or by debates aiming to fine-tune our understanding of connections between human and animal communication. Considerations of both the similarities and the differences between the latter have also been fueled by a flourishing interest for the evolutionary origins of human language.

Here, we contend that much of the discussion on the similarities and differences between human and non-human communication systems also lies within the theoretical positioning on emotions. While in humans, the co-occurrence of a meaningful, semantic aspect and an emotional dimension is not controversial, with communication considered inseparable from emotion (Reilly & Seibert, 2003), in animals, much of the research has so far aimed to determine whether animal communication exclusively reflects affect or meaning (e.g., Wheeler & Fischer, 2012). In the vocal domain, the vocalizations of animals can be affected by emotions, for example affective bursts or rough vocalizations without clear temporal structure. However, this is also the case in human nonverbal sounds such as laughter or cries. Additionally, the prevalence of affect in human communication has naturally led humans to devise ways of conveying emotion even when they engage with each other indirectly, through means of a computer. The aim of this review is to provide an overview of the state of knowledge on nonverbal vocal/auditory communication and emotions in animals and humans, as well as nonverbal emotion in the more recent (written) CMC forms in humans. Overall, we aim to compare these three fields to highlight common ground and areas for future research by uniting them through a common golden thread of affect.

## How Emotion Is Understood Within the Three Fields

We start by summarizing definitions across the fields of animal communication, human communication and CMC, which overlap to some extent. Interest in the study of animal emotions increased exponentially around 30 years ago, propelled by the needs of the pharmaceutical industry (e.g., treatment of human disorders), comparative neuroscience (e.g., development of animal models of human neurological disorders) and animal welfare (Fraser, 2009; Panksepp, 2011). Nowadays, several frameworks allow studying animal emotions (e.g., Désiré et al., 2002; Mendl et al., 2010), encompassing the four components of emotions accessible in these species, along with their indicators and the tools to measure them: neural (e.g., brain activity), peripheral physiological (e.g., heart rate, stress hormones), cognitive (e.g., cognitive bias), and behavioural (e.g., body postures, facial and vocal expressions) indicators (Kremer et al., 2020). This focus on four components excludes the generally accepted fifth component of emotion accessible in humans, that is, the subjective, conscious component (Sander, 2013). The frameworks developed for studying animal emotions have been adapted from human psychology to animals: appraisal theories (Ellsworth & Scherer, 2003) suggest that discrete or modal emotions, arise as a function of specific features of the situation and how the animal appraises them (Désiré et al., 2002). By contrast, the two-dimensional framework (Russell, 1980) suggests that emotions can be mapped according to their arousal (bodily excitation) and valence (positive vs. negative), which can be assessed based on the pleasant (rewarding/attractive) or unpleasant (punishing/repulsive) nature of the situation triggering the emotion (Mendl et al., 2010).

Animal emotions are commonly described as relatively short-term reactions to an external or internal stimulus or event of importance for the organism, characterized by coordinated neural, physiological, cognitive and behavioral changes (Paul & Mendl, 2018). In the absence of clear evidence for feelings, the term ‘emotion’ has been used to refer to emotional-like processing, independently of the degree to which they are consciously experienced (Paul et al., 2020). The more general term of ‘affective states’ encompasses short and longer-term states (e.g., ‘moods’) that are valenced. Emotions and moods play an important role for animal survival: emotions guide responses to stimuli, while moods inform about expectations in the environment (Mendl et al., 2010). By contrast, motivation states reflect the likelihood of performing a given behaviour, or the force that drives this behaviour (‘drives’ or ‘wants’; Dawkins, 2008). Motivation is strongly influenced by underlying affective states, but considered as a distinct phenomenon (Gygax, 2017).

In the field of human affective science, different kinds of affective phenomena have also been described in the literature over the last 30 years (e.g., Sander, 2013). In a multi-componential perspective, emotions are concomitant modifications or synchronizations among different subcomponents of the organisms. These include cognition, in which appraisal is a key component (e.g., appraisal of relevance, implication, causality, coping potential, and norms); the peripheral physiological response, such as respiration, etc.; and expressive behaviours, including vocal or gestural channels. The remaining subcomponents include motivation, in which the concept of action tendencies (i.e. the ‘internal motive states that are hypothesized to underlie a felt urge […]’; Frijda, 2009; Sander et al., 2018, p. 223) is central; and feeling, which is conceptualized as an integrator and monitor, conscious component of emotion (Grandjean et al., 2008; Sander et al., 2018; Scherer, 1984). In this perspective, the main categories conceptualized as basic emotions in an evolutionary discrete perspective (Ekman, 1992), including happiness, fear, sadness, disgust, anger, and surprise are theorized as modal emotions. Beyond these five or six modal emotions, that is, the most often observed emotions and subject of explicit discrete categorization, the multi-componential perspective proposes that the appraisal of a situation or an event can induce an infinite variety of emotions. Note that appraisal can be effortful and conscious, such as in the case of an explicit evaluation of causality, but can also occur at more basic levels, such as overlearned cognitive scripts or habits at schematic or sensorimotor levels (Leventhal & Scherer, 1987).

In the field of CMC, at least with regards to its historically predominant text-based modality, emotions are most often conceptualized as content that can be shared, expressed in a more or less explicit fashion by the sender of a message and recognized or inferred by the recipient of a message (Derks et al., 2008). The preference for this viewpoint, as opposed notably to the construal of emotion as a component of human experience, may have been partly motivated by the observation that the expression of emotional content in CMC is at least separated from the corresponding emotional experience by a number of technological steps, and sometimes does not even correspond to an actual emotional experience. Regardless of the motivation for the field's prevalent conceptual perspective on emotion, a defining characteristic of the overall direction of research in this domain is the gradual shift from a paradigm initially centered on the methods of psychology and sociology to one that emphasizes the methods of data science and corpus linguistics, with researchers taking advantage of the very large datasets generated by social network services. The strong commitment to empirical methodologies has made it all the more necessary to rely on formal models of emotion, chief among which are Ekman's basic categories and various instances of dimensional representations that minimally involve valence and arousal and sometimes additional features such as dominance or surprise (Wood et al., 2018).

## Summary of Approach to Emotions Across Fields and Structure of the Article

**Figure 1.**
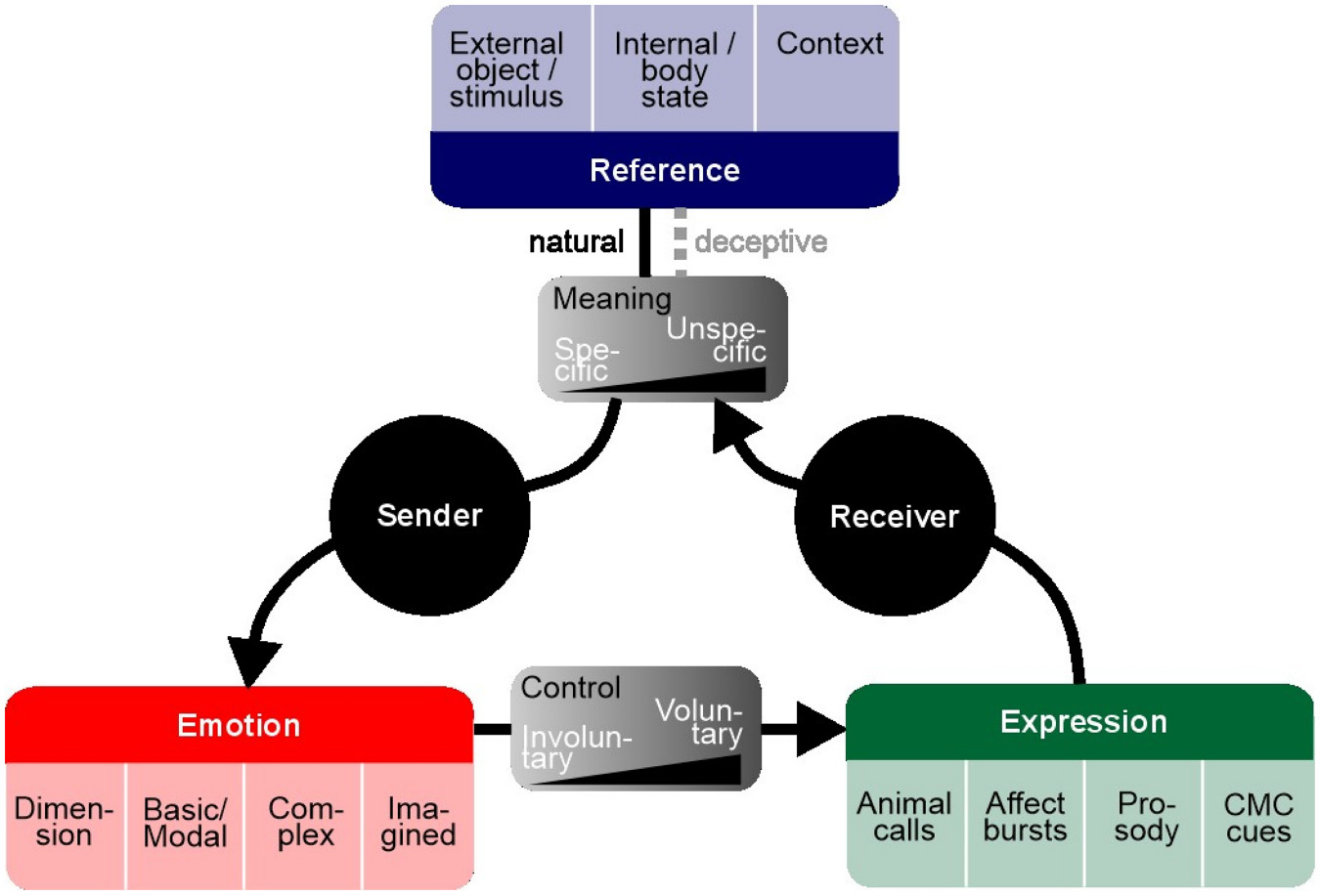
One way to understand nonverbal emotional communication is that it involves a sender and a receiver sharing emotional information and establishing shared knowledge about an important external, internal, or contextual state. The sender (left side) experiences some emotional state triggered by a reference state in cases of naturally occurring emotional expressions. Exceptions are deceptive emotional expressions, which only have a weak or no link to reference states. This reference state (top box) can be a variety of objects, providing a meaningful background for the experience and expression of the emotional state. Meaning can be either specific (e.g., signalling the presence of a specific predator type) or unspecific (e.g., the situation or context is frightening without reference to a specific object). The emotional state of the sender (left box) can vary from basic/simple states to more complex and mixed emotional states (see text for description). Emotional states in the sender eventually lead to nonverbal expressions, expressed more or less voluntarily. The nonverbal expressions (right box) can be of various nature and modality across species and communication tools. The receiver (right side) aims to decode the emotional information encoded in the sender's expressions, potentially by mirroring some of the emotional state of the sender. Successful communication happens when a shared meaning is established between the sender and the receiver, and when a receiver mistakes deceptive emotional signals by the sender is being truthful. Unsuccessful communication happens, when the sender and received disagree on a shared meaning, such in the case of conflicting interactions.

Overall, in our view, there are both similarities and differences in the way emotions have been dealt with in the three domains (Table S1; Figure 1). Animal emotion research remains at a disadvantage, by having to build upon theoretical frameworks inspired by human research and because of humans’ unrivalled use of technology that can decouple emotion from the media. Yet, we will assume here that some aspects of emotion, such as the appraisal aspect, are present in some forms in other species (Désiré et al., 2002). It is worth noting that these appraisals can be implemented at different levels (Leventhal & Scherer, 1987), including aspects that cannot be made explicit, even in humans. Conversely, we contend that the sometimes too-cognitively loaded approach to emotion applied to humans might not be necessary (see below for discussion).

Finally, while we acknowledge that an affective dimension can be found in various other aspects of communication, such as within the choice of words themselves or with a different means of communication (e.g., gestures, Heesen et al., 2022), our review will be solely concerned with nonverbal emotional communication. In the following sections, we will first introduce the nonverbal communication of emotions across the fields before looking at specific aspects that have shaped the discussion over the last few decades. We will discuss the relationship between emotions and control, often addressed within the general debate on intentionality (Townsend et al., 2017); and between emotions and meaning of nonverbal utterances in all three fields of research, using the same order in each section: animal vocalizations, human vocalizations, and CMC. Finally, we will conclude by underlining the similarities and differences across fields and delineate a common work plan across fields to progress in our understanding of the co-occurrence of affect and communication.

## Nonverbal Communication of Emotions

### Nonverbal Communication of Emotions in Animal Vocalizations

Vocal expression of emotions in animals, as well as the perception of these vocal cues by conspecifics, has been revealed in many species (Briefer, 2012, 2018). According to the ‘motivation-structural rules’, the features of bird and mammal vocalization vary in a predictable way depending on the characteristics of the context as fearful (high-frequency and tonal sounds), friendly (soft, low-frequency, amplitude-modulated, and rhythmic sounds) or aggressive/hostile (low-frequency, loud, and noisy sounds) (August & Anderson, 1987; Morton, 1977). In addition, there are similarities in how different species express emotional arousal and valence (Briefer, 2012, 2020); in most species, situations of opposite valence are characterized by the production of different call types (i.e., functionally and acoustically distinct units), while changes in arousal or motivation result in more subtle modifications of the acoustic structure of the sounds (increase in amplitude, rate and frequencies) (Briefer, 2012; Manser, 2010). However, some call types (e.g., contact calls) can also be produced in both positive (e.g., social reunion, foraging) and negative contexts (e.g., social separation, isolation), in which case their acoustic structure changes, as shown for instance in many ungulates (e.g., domestic and wild horses or pigs goats, sheep and cows), with, often, shorter durations, and lower and less variable frequencies in positive contexts (Briefer, 2020).

Research in the field of animal communication has, in recent years, mainly focused on the expression of emotional context, arousal or valence, as well as the discrimination of these sounds and perception/contagion by receivers (see Briefer, 2012, 2018; Scheiner & Fischer, 2011; Zimmermann et al., 2013 for reviews). More recently, expression and perception of emotions across species has been increasingly studied, to investigate the evolution of vocal expression of emotion and test the hypothesis that expression of arousal and maybe also valence has been conserved throughout evolution (Belin et al., 2008; Filippi et al., 2017; Maigrot et al., 2022; Smith et al., 2018). Some work has also focussed on variation within taxonomic families, highlighting both similarities and striking differences in how domestic and Przewalski's horses express emotions (Briefer et al., 2015; Maigrot et al., 2017); and how domestic pigs and wild boars do so (Briefer et al., 2019; Maigrot et al., 2018). For instance, wild boars produce grunts with lower formants in positive than negative contexts, while the opposite is true for domestic pigs (Briefer et al., 2019; Maigrot et al., 2018). Both comparisons have revealed that some acoustic parameters are used in the same way by both species to encode emotional valence (e.g., duration is longer in negative than positive valence in both pigs and wild boars), while other parameters change in opposite directions (e.g., formants are higher in positive compared to negative contexts in pigs, while the opposite occurs in wild boars). By contrast, within species variation in vocal emotion expression has, to our knowledge, not been explored yet in other animals. Such work could be done by, for example, comparing vocal expression of emotions between wild populations or between domestic breeds (e.g., Papadaki et al., 2021). In humans, testing the hypothesis of universality of emotions across different cultures has a long history, for example in the cases of facial (e.g., Ekman & Friesen, 1971) or vocal recognition (Sauter et al., 2010b), whereby recent studies have highlighted significant differences between cultures regarding facial expression (Jack et al., 2012), or the desired expressivity, intensity, and preferred emotion during infant emotion expression (Bard et al., 2021). From our point of view, and as was done in the latter study, the universality of emotions could also be studied in a range of animals. Finally, an increasing amount of work is aimed at deciphering how emotional and referential information is integrated in animal signals, as well as comparing emotional and intentional communication (Price et al., 2015; Seyfarth & Cheney, 2003; Slocombe & Zuberbühler, 2007) as will be discussed in the relevant sections on reference and control.

### Nonverbal Communication of Emotions in Human Vocalizations

Humans can experience a broad variety of basic and complex emotions, and these emotional states influence human behaviour and expressive signals that are used for communicating these emotions to other individuals and to influence the behaviour of conspecifics. Researchers have introduced the concept of emotional prosody to account for situations where emotions have an impact on vocalizations, specifically by acting on respiration, phonation, or articulation (e.g., Grandjean et al., 2006). Indeed, during an emotional episode, the production of vocalizations is modified and the information provided by the speaker, voluntarily or involuntarily, can be used by the listener to infer the speaker's emotional state and then to adjust their behaviour. In this view, vocal production can be characterized by different physical acoustic parameters such as the fundamental frequency (f0), which corresponds at the perceptual level to the pitch; the energy related to loudness; and the spectral components referring to the voice quality. Emotional contents are characterized by different patterns of acoustic parameters (Banse & Scherer, 1996; Sauter et al., 2010b) and those can be used by the listener, through their perceptual correspondence, to infer the emotional state of the speaker.

An important topic of discussion for nonverbal human expression of emotions concerns their effectiveness, particularly the acoustic distinctiveness of expressing emotions in this channel. Previous research has shown that variation in underlying emotions results in largely distinctive vocal nonverbal expressions (Frühholz et al., 2021; Patel et al., 2011; Sauter et al., 2010a). The acoustic distinctiveness however is not exclusive, as there is also acoustic overlap and confusion between some of these vocal expressions, such as fear sharing features with achievement and anger (Sauter et al., 2010a), or intense joy sharing features with panic fear (Patel et al., 2011). The question of the effectiveness has a second component related to how well listeners can detect and recognize the emotions portrayed in these various nonverbal expressions. Again, listeners distinguish and classify the emotions expressed on nonverbal vocal emotions, usually well above chance level (Lima et al., 2019; Sauter et al., 2010a) and across cultures (Sauter et al., 2010b). However, some confusions also occur in listeners while classifying such vocal emotions, such as misclassifying surprise as disgust or relief, anger as disgust or fear (Sauter et al., 2010a), or misclassifying fear and amusement vocalizations (Lima et al., 2019). Such data both support (by allowing classification) and confront (because of the overlap) the basic model of emotions (Ekman, 1992). A consensual view, which we endorse, would be that emotion recognition is mostly a multi-modal process, in that ambiguous vocal expression can be disambiguated by additional sensory information from other modalities (Thorstenson et al., 2022).

Other topics of discussion concern the effect of intensity variations in nonverbal expressions ; the correspondence between acted (‘acted’, ‘play-acted’, or ‘posed’ mainly refers to the case that a person is not in an emotional state, but expresses and pretends an emotion as-if being in an emotional state, they are almost always of a voluntary nature (see Jürgens et al., 2011; Serrat et al., 2020) and spontaneous nonverbal emotion expression; and the sensitivity of certain brain systems for emotional vocal expressions. However, we will not cover these aspects in our review.

### Nonverbal Communication of Emotions in Text Messages

Besides vocalizations, human face-to-face (F2F) communication uses various nonverbal or paralinguistic means to express socio-emotional content, including facial expressions, gestures, or physical proximity. Such cues are obviously missing in CMC, at least as far as its text-based modality is concerned. For this reason, early research in this field typically adopted the ‘cues filtered-out’ perspective, whereby CMC was perceived as a defective channel to convey socio-emotional content (Short et al., 1976). It was not long, however, before it was recognized that CMC users find ways to circumvent the channel's limitations and take advantage of its possibilities to fulfill their communicative needs (Derks et al., 2008; Rice & Love, 1987; Walther, 1992), leading CMC, given sufficient time, to become just as effective as F2F as a means of conveying socio-emotional and relational content—and even more effective in certain circumstances (Walther, 1996). For example, the expression of negative emotions appears to be facilitated by the reduced social presence and visibility that are characteristics of CMC (Derks et al., 2008).

Over time, a wide array of new communicative devices has emerged in CMC, which function as substitutes for F2F paralinguistic cues. The earliest of these paralinguistic devices rely on orthographic and typographic conventions that were already well identified four decades ago: non-standard uses of punctuation and other symbols such as asterisks, parentheses and blanks, non-standard word spellings, capitalization, interjections, acronyms, parenthetical tone or mood descriptions, and so on (Carey, 1980).^1^ In the early 1980s, the emergence of emoticons (character sequences such as ‘:-)’, representing various facial expressions) inaugurated a trend that would gain increasing traction until the present day, namely the conversational use of various types of graphical devices in CMC, also known as ‘graphicons’ (Herring & Dainas, 2017): these include emoticons, emojis, animojis, stickers, GIFs, images, and videos, most of which have become part of CMC as a result of successive technological advances. In this review, we will only be concerned with those graphicons that are encoded by textual means, that is, emoticons (sequences of symbols and punctuation signs usually representing facial expressions) and emojis (graphical symbols such as 
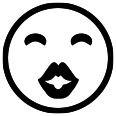
 or 
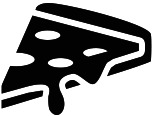
, representing facial expressions, gestures, objects, concepts, etc.). Emojis are by far the most frequently used nonverbal cues at the time of writing (Dürscheid & Siever, 2017; Riordan & Kreuz, 2010), and evidence suggests that they have taken over several communicative functions previously assigned to other types of nonverbal cues (Pavalanathan & Eisenstein, 2016). Yet we believe that the study of other graphicon types will become increasingly important for understanding the evolution of CMC.

### Summary of Nonverbal Communication of Emotion

Overall, in our view, the current trend of research in the three domains has mostly been characterizing how we communicate and understand emotions as different species (Table S1). There are some unquestionable similarities such as the reliance on various physical properties of the sound that allow conveying emotion, although the development of new technologies has also made humans innovate with the goal to convey emotions more clearly. The displayed flexibility with respect to emotion is crucial and may represent a major evolution in the human emotional communication systems, as can be evidenced in two fundamental aspects of communication: control and meaning.

## Control in Emotional Communication

### Control in Animal Vocalizations

There is growing evidence that the level of control animals have over their vocalizations is a continuum; at a basic stage, simple control over respiration rate can affect the rate of call production; at a second stage, control over the respiratory pressure allows modification of the amplitude over time; then, muscle tension control enables changes in frequency of the sounds produced; at the latest stage, a high and direct cerebral forebrain control over vocal production can lead to a refinement of the sounds. Control can also consist in inhibiting sound production. Overall, as the level of cerebral control increases, vocal production becomes less dependent on the physiological state (and hence emotion) of the producer, and prone to voluntary manipulations (Tchernichovski & Oller, 2016). This can result, amongst others, in vocal imitation (or ‘complex vocal learning’; that is, the ability to produce entirely new sounds by imitation) (Tchernichovski & Oller, 2016; Tyack, 2020). Other than in humans, such ability for imitation has been found so far only in three vertebrate groups: birds (songbirds, hummingbirds and parrots), nonhuman primates, and a few nonprimate mammals amongst cetaceans, pinnipeds, elephants and bats (Lattenkamp & Vernes, 2018). Since most species have relatively less control over their vocalizations compared to humans (Jürgens, 2009), emotions are expected to influence vocal production in animals even more directly than in humans, whose voice features depend, notably, on socio-cultural and linguistic conventions as well as additional intentional manipulations (Scheiner & Fischer, 2011).

The topic of control in animal vocalizations has been intertwined with the long-standing debate on intentional (goal-directed) communication in non-humans (Marler et al., 1992; Sievers et al., 2017; Townsend et al., 2017). While animal scientists have strived to adopt a common position to isolate criteria of intentional production in vocal communication (Townsend et al., 2017), echoing some of the earlier work in gestural communication (Genty et al., 2009; Liebal et al., 2014), much of this theoretical framework falls short of the high-cognitive load required for human intentional communication (Scott-Phillips, 2015), leading to possible irreconcilable difference with human communication (Tomasello, 2008). Yet, animals display a surprising flexibility in call production, including the so-called emotional ones (Sievers et al., 2017). For example, victims of aggressions in chimpanzees vary the acoustic structure of their screams depending on the severity of the aggression they are facing, but also according to the audience, suggesting that they strategically modify their calling pattern to recruit help (Slocombe & Zuberbühler, 2007). This example outlines the broader ability of a number of animal species to engage in deception, which can be found in many forms, including the manipulation of vocal production (Byrne & Whiten, 1988). Overall, beyond manipulation, the ability to refrain from or produce ‘on demand’ calls, as when engaged in sexual intercourse with desirable individuals (Clay et al., 2011), underlines the inaccurate classification of animal calls as pure emotional reactions produced without any control (Tomasello, 2008). This is especially the case if animals are able to exert some control on their vocal production in some of the most stressful contexts (see also Crockford et al., 2012 in a predation context). We note that most of the examples in this section come from primates; although efforts are now underway to shift the lens from a generally primate-centric field (Nieder & Mooney, 2020; Ravignani & Garcia, 2022; Townsend et al., 2012), we acknowledge that not all animal species will have the cognitive flexibility to exert such control on their vocal production. This however does not change the fundamental message of this section, which is that emotions must be seen as a dimension of animal communication, which interacts with control, rather than as a killjoy factor that precludes any control (see also Kret et al., 2020; Sievers & Gruber, 2020).

### Control in Human Emotional Vocalizations

In the literature on humans, researchers are more concerned with the controlled expression of emotion through speech, rather than with the intentional production of emotional calls, a notable difference with the animal literature. Humans have two major nonverbal vocal channels to express and communicate emotions (Frühholz & Schweinberger, 2021). The first channel seems evolutionarily older and shared with many mammalian, and other vertebrate species. This channel refers to nonverbal expressions of vocal emotions, and is typically used to express basic emotional states (Patel et al., 2011; Sauter et al., 2010a). Emotions expressed this way can be short ‘affective bursts’ (Scherer, 1994) and are usually defined as basic emotional states triggered by perceptual and mental experiences that only involve some low-to-medium level of cognitive processing and evaluation. Given that these nonverbal affective bursts are usually triggered by external and internal cues, the level of control in these bursts is rather low. However, the expression of these nonverbal affective bursts can be controlled to some degree, if required by certain contexts. The expression of these bursts can be inhibited, delayed, or attenuated. Yet, this requires a high level of top-down control and emotion regulation, as well as a certain level of ontogenetic development according to some researchers (Barrett, 1993). Overall, this channel seems sometimes processed faster with regard to accuracy of recognition of the expressed emotion in listeners, compared to the second channel (Frühholz & Schweinberger, 2021).

This second channel refers to vocal modulations of speech utterances, which are referred to as emotional prosody. Thus, while humans express emotional information in their speech utterances based on linguistic rules, they simultaneously also express their emotions in the paralinguistic channel of prosodic voice modulations. This paralinguistic channel is used to express both rather basic emotions and more complex emotions specific to human interactions (Alba-Ferrara et al., 2011).

Further topics concern the commonalities and differences between spontaneous and acted nonverbal vocal emotions (Anikin & Lima, 2018; Bryant et al., 2018; Engelberg & Gouzoules, 2019; Jürgens et al., 2011; McGettigan et al., 2015). Again, there is overlap as well as distinctiveness between spontaneous and acted nonverbal emotions, both in acoustic and in perceptual terms. Listeners can distinguish spontaneous from acted vocalizations above chance level (∼56%–69%) (Anikin & Lima, 2018; Bryant et al., 2018), but this rather low detection rate indicates a confusion between both vocalization types, pointing to an acoustic similarity between them (Anikin & Lima, 2018; Engelberg & Gouzoules, 2019).

### Control in Text Messages

Emotion expression in CMC is strongly controlled. When questioned about it, respondents appear to have formed a reasonably clear representation of how and why they and others use nonverbal cues in CMC (Gullberg, 2016; Kelly & Watts, 2015). Some linguistic aspects of verbal communication are known to be related (in a presumably mostly non-intentional way) to affective states, in particular intensity, immediacy, and diversity (Bradac et al., 1979). The few studies that have investigated whether and how these relations extend to nonverbal cues in CMC have mostly focused on the receivers’ perception and behavioural response to variations of these parameters in messages (Andersen & Blackburn, 2004; Harris & Paradice, 2007; Wang, 2003). Further research concerning the relationship between the affective and general mental state of the sender and the patterns of uses of nonverbal cues in their messages must follow.

Much like the biological properties of a species’ vocal tract shape the space of possible vocalizations, the technological infrastructure that is inseparable from CMC places strong constraints on the form that communication acts can take in this context, and thus on the variation space over which control may theoretically be exerted by users. For instance, the number of available emojis has grown from 90 at their creation in the late 1990s to more than 3,000 at the time of writing, making the selection of an emoji a very different and in principle much more informative decision on the part of the sender of a message. In practice however, emoji distributions observed in large CMC datasets exhibit Zipf-like properties, in that a limited subset of them accounts for a large proportion of occurrences (Ljubešić & Fišer, 2016; Lu et al., 2016). It is also worth noting that certain cues, notably emojis, may be rendered differently on the receivers’ devices than on the sender's one, effectively subtracting to the latter's potential degree of control on their communication and increasing the potential for misconstrual on the receiver's part (Miller et al., 2016; Shurick & Daniel, 2020; Tigwell & Flatla, 2016).

Recent years have witnessed a gradual increase in the amount of algorithmic intervention during the preparation of a message, which also contributes to lessen the sender's degree of control. This is notably the case of automatic correction and predictive typing, whereby an algorithm suggests the most likely completion of a word based on what the user has previously typed. This has resulted in a rarefaction of non-standard forms such as those involving abbreviation or letter repetition, since they now require a deliberate effort to include, in contrast to the efficiency concerns that initially motivated their use (Herring, 2019). It is likely that the application of similar technologies for suggesting the replacement of words or phrases by specific emojis has fostered the proliferation and diversification of emojis. This hypothesis underlines the relevance of technological factors, which are at least partly outside of the users’ control, for explaining large-scale trends observable in the evolution of CMC data. More advanced technologies leveraging artificial intelligence methods (e.g., automatic translation and so-called ‘smart replies’, that is, entire predefined answers automatically suggested to the user) have emerged recently and little is known at this point about how often they are used and how strongly they influence CMC practices, which makes them an important stake for future research (Hancock et al., 2020).

### Summary of Control in Emotional Communication

Overall, in our view, intriguing parallels can be drawn from the different literatures (Table S1). While the human emotion literature can freely investigate differences between acted or spontaneous production, both the animal literature and the CMC literature are presented with challenges to study the general propensity of the senders to control what they express. For animals, this is part of a long-standing debate on intentionality, while for CMC, there has been little research on the emotional state of the sender and how it affects how they produce messages. We contend that the research in the three fields can meaningfully influence each other with the debate on intentional production being especially interesting for CMC research. Conversely, how CMC research studies the progressive insaturation of a lack of control by predictive typing, or the involuntary misconstrual of a signal can influence how animal and human researchers assess the production and perception of emotional signals in their study systems.

## Meaning in Emotional Signals

### Meaning in Animal Vocalizations

The notion of ‘meaning’ in animal communication is highly debated (Scarantino & Clay, 2015; Wheeler & Fischer, 2012). One can differentiate between two types of meaning; ‘literal meaning’, which is the code that links sounds to what they represent (e.g., referent), and ‘intended meaning’, which requires an understanding of the signaller's intention within a social context (Grice, 1957). In animals, it might be challenging to differentiate between these two types of meaning, as it requires knowledge about whether signals are intentionally produced or not, which we addressed above, and whether the signalling animal displays theory of mind abilities. However, most researchers in the field would agree that, whether or not animal vocalizations are voluntarily produced or intentional, they provide listeners with ‘information’ in the sense that they reduce uncertainty (e.g., about upcoming social interactions or events in the environment) (Marler, 1961; Seyfarth & Cheney, 2017). The information acquired by receivers will depend both on the properties of the signal and the context of production (Seyfarth & Cheney, 2017).

The best example of ‘meaning’ in animal vocal communication can be found in alarm calls that map onto predators. The discovery of alarm calls in vervet monkeys (*Chlorocebus pygerythrus*), which vary according to the types of predators that are approaching (i.e., refer to an event external to the producer) represented a landmark finding in animal communication (Seyfarth et al., 1980). These signals, later termed ‘functionally referential calls’ (Marler et al., 1992) (hereafter ‘FRCs’) provide very specific information about external objects or events to receivers. According to Macedonia & Evans (1993), to define a vocal signal as referential, the following criteria need to be met; production needs to be context-specific (i.e., linked to the presence of a particular external referent; ‘production specificity’), and appropriate responses to the calls need to be stimulus-independent (i.e., the signal itself needs to elicit an appropriate response even in the absence of the referent; ‘perception specificity’). These calls have now been identified in the repertoire of several species and can refer to predators, food or social interactions (Townsend & Manser, 2013).

FRCs raised the idea that animal vocalizations might refer not only to the internal state of the producer, but also specifically to external referents. The semantic aspect of FRCs from the producer's side (i.e., production mechanism) is highly debated (Scarantino & Clay, 2015; Seyfarth & Cheney, 2003; Townsend & Manser, 2013; Wheeler & Fischer, 2012). Some researchers have argued that signals can be purely emotional in their production and still meet the criteria of FRCs, if they are linked in a predictable way to the presence of an external referent (i.e., through a tight association between the presence of a referent and the internal state of the producer). In this case, receivers should still be able to extract specific meaning from the signal regarding the context of production, hence fulfilling the perception specificity criterion (Seyfarth & Cheney, 2003; Wheeler & Fischer, 2012).

Nevertheless, the most parsimonious position argues against a strong dichotomy between emotionality and referentiality. For example, the description of the meerkat (*Suricata suricatta*) alarm call system shed new light on the study of referential and emotional signalling, since the alarm calls of this species vary as a function of both the type of predator approaching and the level of urgency for each predator type (Manser, 2001). It thus suggests that animal vocalizations can simultaneously contain referential and emotional components, similar to human speech (Manser et al., 2002; Scherer, 2003). These two components could be either encoded in different vocal parameters (‘segregation of information’ (Marler, 1960)) or in the same component, in which case they might interact.

Overall the question of whether producers of FRCs are referring to an external stimulus in the same way as human semantic communication, as well as the convergence of the debates between intentional production and reference (see Sievers & Gruber, 2016), remain crucial questions regarding the evolution of language (Christiansen & Kirby, 2003; Fitch, 2010). Yet, one may also acknowledge that the strong dichotomy between emotion and meaning applied to animal vocalizations does not subject them to the same standards as human speech, where affect is often complementary to, and part of, meaning.

### Meaning in Human Emotional Vocalizations

There are typical nonverbal expressions of emotions, such that humans vocally cry when experiencing states of sadness, they laugh when being overwhelmed by joy (Scott et al., 2014; Szameitat et al., 2009), they angrily growl when being aggressive towards natural or social obstacles (Raine et al., 2019), or they fearfully scream when being terrified by an external threat (Arnal et al., 2015; Frühholz et al., 2021). Although humans can express a variety of emotional states in nonverbal expressions, their exact number, and whether they refer to basic emotional states or more complex emotions is debated. Previous reports differ on the number of different nonverbal vocal emotions reported, ranging approximately from 8 (Lima et al., 2013) to 18 (Bänziger & Scherer, 2010). Recent reports also described subtypes of vocal expressions, such as for positive nonverbal expressions (e.g., happiness, amusement, interest, relief, etc.) (Kamiloğlu et al., 2020) or for different types of scream calls (Frühholz et al., 2021).

In terms of the expressive component, most of these basic expressions of emotions would be classified as a ‘symptom’ in semiotic terms (Frühholz et al., 2016), given that the primary reference is to the emotional state of the speaker. This nonverbal expression, however, could also figure as a ‘signal’ or ‘index’ when related to their communicative component to trigger certain responses in listeners. An aggressive growl, for example, can be a symptom of the inner angry state of a person, and it signals another person to immediately distance from the aggressor. In general, the meaning of nonverbal expressions both related to expression and communicating an emotional state is relatively non-arbitrary, given their high acoustic distinctiveness and perceptual recognizability (Frühholz et al., 2021). However, nonverbal expressions in humans seem to be more arbitrary than similar expression in animals, since the reference to external objects (e.g., a scream signals potential danger) is often ambiguous (e.g., a scream does not signify the exact type of danger) and can only be disambiguated by further sensory information (e.g., visual search to identify the source of danger).

In humans, language also allows the transmission of linguistic information, rooted in segmental units (phonemes, syllables, words), which will allow the communication of concepts or facts based on semantic representations (e.g., Friederici, 2005). Moreover, emotions can also be the object of semantic representations and discourses that are used for example in interpersonal emotional regulation (Braunstein et al., 2017). Information at the linguistic and semantic levels can also be in conflict with the information transmitted by the emotional prosody (Schwartz & Pell, 2012). For example, sarcasm or irony is a phenomena referring to an antagonistic tension between semantic aspects (e.g., negative content) and supra-segmental emotional information (positive emotional prosody, see Cheang & Pell, 2008). The context in which these complex utterances are produced is also central in understanding the ironic or sarcastic character of a statement.

### Meaning of Nonverbal Cues in Text Messages

From the point of view of meaning, a defining feature of the most frequent types of CMC nonverbal cues is that they are signs whose meaning refers to another sign, namely an F2F nonverbal cue. This is for example the case of emojis referring to facial expressions (Dürscheid & Siever, 2017) or gestures, but also of several other, older types of cues such as letter repetitions and capitalizations, typically referring to voice quality alterations. Different types of CMC cues appear to specialize in the reference to certain F2F cue types, but there is also a certain degree of overlap: for instance, both emojis and emoticons can represent facial expressions, which opens possibilities for users in terms of identity display and in- or out-group marking. There are of course many nonverbal CMC cues that do not refer to F2F nonverbal cues, some of which carry a clear emotional load, but most of them are considerably less frequent than the aforementioned cues, the main exception to this observation being the 
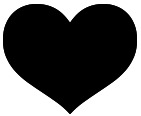
 emoji and its variants.

Concerning emojis in particular, significant differences in their use have been observed across countries (Lu et al., 2016), but their interpretation seems relatively consistent across languages (Barbieri et al., 2016a; Novak et al., 2015). It does however vary according to a range of factors, chief among which are socio-demographic features of users (notably gender, see e.g., Oleszkiewicz et al., 2017; Prada et al., 2018), the specifics of the communication context (notably co-occurring verbal and nonverbal emotional cues, see Vandergriff, 2013), and the considered platforms and media (Tauch & Kanjo, 2016). Emoji interpretation also varies according to the users’ opinions on their use and what they individually represent, creating ambiguity and misunderstandings, as context does not significantly reduce the latter (Miller et al., 2016, 2017). With time and repetition, emojis can also take on idiosyncratic meanings whose interpretation is unique in a certain group or relationship (Al Rashdi, 2015; Gullberg, 2016; Kelly & Watts, 2015; Wiseman & Gould, 2018), similarly to their F2F counterparts such as hand gestures.

The continuous accumulation of vast amounts of written CMC data over the last few decades makes it possible to use data science methods to not only attempt to characterize the meaning (emotional or not) of nonverbal cues at a large scale (Barbieri et al., 2016b; Novak et al., 2015), but even to trace their evolution over time. Applying these techniques to emojis in Twitter data spanning 6 years – a sizable portion of the history of emojis since emergence at the worldwide scale – Robertson et al. (2021) show that the meaning of most emojis is relatively stable over time, with a small subset of them undergoing more considerable semantic change, with emojis with more concrete meanings more likely to undergo semantic change. A major challenge for future research is to move away from data gathered on those web services that make them most readily available, and to adapt data-analytic methodologies to CMC contexts that may be more representative of F2F communication practices, such as instant messaging, for which data are typically not accessible in comparable volumes.

### Summary of Meaning in Nonverbal Emotional Communication

Overall, in our view, the issue of meaning parallels the one of control (Table S1). Once again, research on animals has to contend with the assumption that their signals are fixed, with little flexibility around, with new layers periodically added to the debate such as the presence or absence of characteristic such as arbitrariness (Sievers & Gruber, 2020; Watson et al., 2022). This contrasts strongly with the flexibility displayed by humans in both their vocal non-verbal and CMC communication, where a change of prosody can radically alter the meaning of an expression.

## Conclusion

Looking at the three different communication systems indicates that aspects of emotion, control and meaning, are present in all of them. However, in animals the main communicative part is related to the expression of emotions, whereas in human, it is about emotions and meaning, and in text messages, meaning is the foremost goal and emotions are a secondary addition. As our review has shown, the nonverbal communication of emotion has always been uncontroversial in the case of human vocalizations, where it is widely acknowledged to be an integral part of this means of communication through prosody. In contrast, the study of emotion in nonverbal domains such as animal communication and CMC has required more careful discussion before being established as worthy topics of discussion, and it is only in recent times that researchers embrace emotions as an integral part of animals’ lives (de Waal, 2011), particularly in the context of welfare science, where new research paradigms aim to align with human emotion theory to decipher animal emotions based on nonverbal signals. On the other hand, CMC had to rely on a combination of technological evolutions and innovative practices that progressively allowed the communication of emotion, particularly through the development and use of emoticons and, since the 2010s, emojis, which continuously increase the ability of users to convey their emotions.

As illustrated in our second section, the debate on vocal communication in animals has been fuelled with comparisons with human language properties such as control and intentional production. As with emotional content, human speech is the standard, and the question of control and intentional production remains unquestioned in human vocal production. While some uncontrolled outbursts are produced in specific contexts such as spontaneous crying or laughing, they are connected to basic emotional states, with humans rather described as able to willingly modulate acoustic parameters to express an intended emotion, whether experienced or not. Interestingly, we have seen that CMC could possibly widen the gap with animal communication. Emotion communication in CMC is indeed seen as completely under control, as the user is only limited by the available set of tools at their disposal to transmit the emotion or emotional tone they want to convey. Yet, this has to be modulated by the increasing reliance on automatic algorithmic interventions that may lessen the user's control, as well as the uncontrollable possible differences that may appear on the other user's screen upon reception of the message. In contrast with this human image of emotional communication, intentional production remains largely debated in animal communication, where the application of a human-defined Gricean communication framework imposes large cognitive demands on animals that may not display them. Yet, we have listed several aspects that have shown a not so black-and-white picture in animals, including some control in stressful contexts. Overall, in our view, while human nonverbal communication, whether vocal or through CMC, remains undoubtedly intentional, a certain continuity has been established over the last few decades with animal communication, which is not limited to uncontrolled emotional bursts.

As illustrated in our third section, meaning has also constituted a point of contention when comparing animal and human vocalizations. While the findings of functionally referential signals in non-humans have opened the door for the discussion on less emotionally based signals in animals, they have been framed in an unhelpful opposition between meaning and emotion, largely ignoring more consensual positions that could combine both. Animal emotion research may benefit from human approaches that set the debate in terms of flexibility of use from the producer's side, and flexibility of understanding from the listener's side, rather than in opposing two complementary aspects of vocal communication, as our review of human communication in two modalities (speech and CMC) has shown. Once again, meaning is a defining feature of human communication, making it hard to abstract away from it altogether. Emotional basic signals, such as an aggressive human growl, give contextual information to the listener about the producer. However, the producer may also alter the prosody of an utterance to convey a radically different meaning than indicated by the lexical content (as in a sarcastic congratulation). This intentional and meaningful use of prosody variation adds another layer to the flexibility with which humans can display their emotions. Another way to add on flexibility is to rely on CMC. While the study of CMC has a relatively short history, it is already clear that the meaning attached to emojis remains stable, offering a consistent way to express one's emotions. Yet, specific groups can also attach a specific meaning to a given emoji, allowing a variety of meanings only available to the insiders. Overall, these findings in both human communication and CMC highlight the versatility of human meaningful emotional signals. This very much contrasts with current debates on animal communicative signals, where the discussion is often limited to opposing emotion to meaning.

Overall, we have sought to underline the role of affect in nonverbal communication across species and media of expression. Beyond outlining similarities and differences across domains of research (Table S1), our review also highlights how affect can contribute to bridging research fields that have sometimes remained unconnected because of the methods they use (e.g., CMC), or have diverged because of ideological backgrounds (e.g., animal and human communication). For example, debates on animal and human communication do not accurately reflect the continuity between the two (see also Moore, 2013), and are threatened by an increasingly large gap that isolates the study of human linguistic communication, seen as highly cognitive, from that of other communication systems (e.g., Scott-Phillips, 2015), with little hope of comparing systems. Yet, other models of communication are less cognitively demanding (Moore, 2013; Sievers & Gruber, 2020). As our review has shown, emotions play an integral part in communication and can contribute to bridging different fields of research.

Although methodological limitations may limit comparisons, nonverbal communication needs to be approached from an evolutionary point of view of the requirements for the efficiency of communication systems. As such, we believe that our review allows the drawing of inferences about the evolution of emotion, and how human's harnessing of their emotions as a communicative tool has truly exploded in our lineage, from a last common ancestor with chimpanzees and bonobos possibly closer to the latter two (for a review, see Gruber & Clay, 2016). Our review underlines a pattern where animal signals express mainly the emotional side with limited variation in meaning, whereas in human communication, meaning takes over, without emotions being any less present; finally, CMC started by only including meaning, but emotional expressions were rapidly brought in addition. We develop this further here: animal vocalizations appear to mainly relate to the behavioural context the producer experiences (motivation) and only in very few contexts refer to external events or objects. Animal communication thus remains tightly linked to the emotional state of the producer, and arguably, also of the receiver. While this emotional layer has too often been opposed to the possibility that the calls may nonetheless be meaningful for both the producer and the receiver (e.g., Crockford et al., 2012), and produced as part of an intentional act of communication, the current state of knowledge suggests that our last common ancestor's use of emotions *as a means for* intentional communication remains at most basic. This is in strong contrast to humans, where the large repertoire of signals appears to be frequently related to external events or objects, or temporarily related to the past or future (Figure 1). Yet, the emotional expression here is present in all aspects, and our species has in fact developed ways of harnessing our emotions to intentionally manipulate the message we want to convey. This is valid both for nonverbal vocal communication and for the more recently developed CMC that is taking an increasingly large part of our social lives. While the original goal of CMC was to communicate meaningful information as efficiently as possible by reducing redundant information in the signals used in human speech or written language, a number of paralinguistic cues, chief among which emojis, had to be brought in to avoid ambiguity in communication and reflect the emotional information notably found in prosody. We note that despite our claimed focus on nonverbal communication systems, we could not avoid including other media of communication, such as found during F2F interaction. This is because communication is, at its core, multimodal (Fröhlich et al., 2019), and that it remains difficult to split the contribution of each means of communication. As such, we acknowledge that some of our arguments, particularly pertaining to animal communication, must be assessed contextually, with each medium of communication allowing their own flexibility. For example, the emotional content of animal gestures remains much unknown but can be analysed in a manner similar to how emotional vocalizations are analysed (Heesen et al., 2022). Similarly, humans readily associate facial expression such as smiles with vocalizations (Drahota et al., 2008), making integrating control, meaning and emotion a multimodal endeavour in the future across all three fields.

Overall, the abilities to convey accurate or false emotional information, and to intentionally modify the meaning of an utterance through prosody, both appear to have emerged in our lineage, although how early remains unknown (i.e., did this ability emerge before our mastering of language, or preempted it?). By including CMC, our review illustrates that emotional expression is no longer constrained by our biology. With an ever-increasing reliance on CMC and technology, we expect that our species will find additional ways to intentionally and meaningfully express their emotion relying on cultural rather than biological evolution. Yet our review also suggests that many mechanisms remain similar between the three domains reviewed. Finally, as it is widely found across species, we suggest that emotional conveyance can meaningfully contribute to the more general debates on the evolution of communication, and particularly language, in the human lineage.

## Supplemental Material

sj-docx-1-emr-10.1177_17540739241303505 - Supplemental material for Emotion in Nonverbal Communication: Comparing Animal and Human Vocalizations and Human Text Messages Supplemental material, sj-docx-1-emr-10.1177_17540739241303505 for Emotion in Nonverbal Communication: Comparing Animal and Human Vocalizations and Human Text Messages by T. Gruber, E. F. Briefer, A. Grütter, A. Xanthos, D. Grandjean, M. B. Manser and S. Frühholz in Emotion Review

## Notes

1. Interestingly, the large-scale use of such devices has not been attested in written correspondence, which raises the question whether their emergence in CMC can be partly explained by the fact that this communicative context has a pace which is considerably closer to the rapid back and forth of face-to-face conversations.
