# Supplementary material for: Emotion in Nonverbal Communication: Comparing Animal and Human Vocalizations and Human Text Messages
Source: Emot Rev. 2025 Jan 15;17(1):30–45. doi: 10.1177/17540739241303505 (PMC12161768; doi:10.1177/17540739241303505)
Supplement: sj-docx-1-emr-10.1177_17540739241303505 - Supplemental material for Emotion in Nonverbal Communication: Comparing Animal and Human Vocalizations and Human Text Messages [file sj-docx-1-emr-10.1177_17540739241303505.docx]

**Table S1.** Comparison (convergences and divergences) between the three domains of research (Animal Communication (AC), Human Communication (HC), and Computer-Mediated Communication (CMC)), in relation to the main topics discussed in this review, along with key references.

| **TOPICS** | **AC** | **HC** | **CMC** |
| --- | --- | --- | --- |
| **Definitions of affective states** | - Emotions = short-term, linked to a specific stimulus [1] - Mood = long-term, diffuse, results from the accumulation of short-term emotions [2] | | |
| **Assessment methods** | - Use of frameworks: e.g. two-dimensions, appraisal theories [3] - Use of emotional indicators: neuro-physiological, behavioural (e.g. expressions), cognitive changes [4] | | |
|  |  | - Use of emotion self-report [5] | |
| **Advantages for the study of emotional communication** | - More direct influence (less control) of emotions on vocalizations than HC and CMC [6] - Possibility to compare multiple species living in various and natural environments [7, 8] | - Numerous frameworks [1] - Advanced methods easily available (neuroimaging); direct access to conscious component (feeling) [9] | - Very large datasets easily accessible [10, 11] |
| **Limitations to the study of emotional communication** | - Inferred from situation/indicators (subjective component not accessible) [3] |  |  |
| **Emotion expression** | Two levels of expression:   - Call types (e.g. growl vs bark) - Structure (parameter modulation within a call type) [7, 8] | Three levels of expression:   - Nonverbal expressions (e.g. affective bursts) - Emotional prosody (voice modulation in speech) [12] - Emotional information in speech utterances (e.g. verbal content) | Three levels of expression:   - Word choice and arrangement [13] - Orthographic and typographic conventions [14] - Graphicons (including emojis) [15, 16] |
| **Control** | - Continuum across species: control over call production → modification of amplitude → modification of frequency → vocal imitation [17]   Intentionality in sound production varies between species (e.g. audience effect) [18] | - Control lower in nonverbal expressions of vocal emotions and higher in emotional prosody [19] - Spontaneous vs acted expressions [20] | - High control overall [21, 22] - Constraints imposed by technological infrastructure |
| **Meaning** | - Use of term ‘meaning’ usually restricted to external events (not internal states) [23] - Dichotomy between emotion and meaning [24, 25] | - Meaning can refer to both emotions or external events | |
| **Main research topics** | - Expression of emotional context, arousal or valence   [7, 8]   - Perception/contagion of emotion expressions (neuro-physiological, behavioural and cognitive responses to emotional sounds) [26] - Cross-species expression and perception [27] - Emotional vs referential communication [28] - Emotional vs intentional communication [29] | - Correspondence between acted and spontaneous nonverbal emotion expression [30, 31] - Effect of intensity variations in nonverbal expressions [13] - Sensitivity of certain brain systems for such expressions | - Automatic detection and recognition of emotional content [13] |
|  |  | - Effectiveness in expressing, communicating, and recognizing certain emotions [32] - Comparisons across populations/cultures [16, 31, 33] | |

**References**

1. Sander D. 2013 Models of emotion: the affective neuroscience approach. In *Handbook of Human Affective Neuroscience* (Cambridge, Cambridge University Press.

2. Mendl M., Neville V., Paul E.S. 2022 Bridging the Gap: Human Emotions and Animal Emotions. *Affective Science* **3**(4), 703-712. (doi:10.1007/s42761-022-00125-6).

3. Mendl M., Burman O.H.P., Paul E.S. 2010 An integrative and functional framework for the study of animal emotion and mood. *Proc R Soc B* **277**, 2895–2904.

4. Kremer L., Holkenborg S.K., Reimert I., Bolhuis J.E., Webb L.E. 2020 The nuts and bolts of animal emotion. *Neurosci Biobehav Rev* **113**, 273–286.

5. Posner J., Russell J.A., Peterson B.S. 2005 The circumplex model of affect: an integrative approach to affective neuroscience, cognitive development, and psychopathology. *Dev Psychopathol* **17**(3), 715-734. (doi:10.1017/s0954579405050340).

6. Jürgens U. 2009 The neural control of vocalization in mammals: a review *J Voice* **23**, 1-10.

7. Briefer E.F. 2012 Vocal expression of emotions in mammals: Mechanisms of production and evidence. *Journal of Zoology* **288**(1), 1-20. (doi:10.1111/j.1469-7998.2012.00920.x).

8. Briefer E.F. 2020 Coding for ‘Dynamic’ Information: Vocal Expression of Emotional Arousal and Valence in Non-human Animals. In *Coding Strategies in Vertebrate Acoustic Communication* (eds. T. Aubin, Mathevon N.), pp. 137–162. Cham, Springer International Publishing.

9. Sander D., Grandjean D., Scherer K.R. 2018 An Appraisal-Driven Componential Approach to the Emotional Brain. *Emotion Review* **10**(3), 219-231. (doi:10.1177/1754073918765653).

10. Barbieri F., Ronzano F., Saggion H. 2016 What does this Emoji Mean? A Vector Space Skip-Gram Model for Twitter Emojis. In *Proceedings of the Tenth International Conference on Language Resources and Evaluation (LREC’16)* (pp. 3967-3972.

11. Novak P.K., Smailović J., Sluban B., Mozetič I. 2015 Sentiment of Emojis. *PLOS ONE* **10**, e0144296.

12. Frühholz S., Trost W., Kotz S.A. 2016 The sound of emotions: Towards a unifying neural network perspective of affective sound processing. *Neurosci Biobehav Rev* **68**, 96-110.

13. Roswandowitz C., Swanborough H., Frühholz S. 2021 Categorizing human vocal signals depends on an integrated auditory-frontal cortical network. *Human Brain Mapping* **42**(5), 1503-1517. (doi:<https://doi.org/10.1002/hbm.25309>).

14. Carey J. 1980 Paralanguage in Computer Mediated Communication. In *18th Annual Meeting of the Association for Computational Linguistics* (pp. 67–69.

15. Ljubešić N., Fišer D. 2016 A Global Analysis of Emoji Usage. In *Proceedings of the 10th Web as Corpus Workshop* (pp. 82–89.

16. Lu X., Ai W., Liu X., Li Q., Wang N., Huang G., Mei Q. 2016 Learning from the ubiquitous language: an empirical analysis of emoji usage of smartphone users. *Proceedings of the 2016 ACM International Joint Conference on Pervasive and Ubiquitous Computing*.

17. Tchernichovski O., Oller D.K. 2016 Vocal development: how marmoset infants express their feelings. *Curr Biol* **26**, R422-R424.

18. Sievers C., Wild M., Gruber T. 2017 Intentionality and flexibility in animal communication In *Routledge Handbook of Philosophy of Animal Minds* (eds. K. Andrews, Beck J.), pp. 333-342. London and New York, Routledge.

19. Owren M.J., Rendall D. 2001 Sound on the rebound: bringing form and function back to the forefront in understanding nonhuman primate vocal signaling. *Evolutionary Anthropology* **10**, 58-71.

20. Jürgens R., Hammerschmidt K., Fischer J. 2011 Authentic and play-acted vocal emotion expressions reveal acoustic differences. *Front Psychol* **2**.

21. Kelly R., Watts L. 2015 Characterising the inventive appropriation of emoji as relationally meaningful in mediated close personal relationships. In *Experiences of Technology Appropriation: Unanticipated Users, Usage, Circumstances, and Design* (Oslo, Norway.

22. Gullberg K. 2016 Laughing face with tears of joy: A study of the production and interpretation of emojis among Swedish University Students, University of Lund, Sweden.

23. Seyfarth R.M., Cheney D.L. 2017 The origin of meaning in animal signals. *Anim Behav* **124**, 339-346.

24. Seyfarth R.M., Cheney D.L. 2003 Meaning and emotion in animal vocalizations. *Emotions inside Out* **1000**, 32-55. (doi:10.1196/annals.1280.004).

25. Sievers C., Gruber T. 2020 Can nonhuman primate signals be arbitrarily meaningful like human words? An affective approach. *Animal Behavior and Cognition*.

26. Briefer E.F. 2018 Vocal contagion of emotions in non-human animals. *Proc R Soc B* **258**, 20172783.

27. Filippi P., Congdon J.V., Hoang J., Bowling D.L., Reber S.A., Pašukonis A., Hoeschele M., Ocklenburg S., de Boer B., Sturdy C.B., et al. 2017 Humans recognize emotional arousal in vocalizations across all classes of terrestrial vertebrates: evidence for acoustic universals. *Proceedings of the Royal Society B: Biological Sciences* **284**(1859). (doi:10.1098/rspb.2017.0990).

28. Townsend S.W., Manser M.B. 2013 Functionally referential communication in mammals: the past, present and the future. *Ethology* **119**, 1-11.

29. Heesen R.M., Sievers C., Gruber T., Clay Z. 2021 Primate Communication: Affective, Intentional, or Both? *OSF Preprints*. (doi:10.31219/osf.io/g5zse).

30. Anikin A., Lima C.F. 2018 Perceptual and acoustic differences between authentic and acted nonverbal emotional vocalizations. *Q J Exp Psychol* **71**, 622-641.

31. Bryant G.A., Fessler D.M.T., Fusaroli R., Clint E., Amir D., Chávez B., Denton K.K., Díaz C., Duran L.T., Fanćovićová J., et al. 2018 The perception of spontaneous and volitional laughter across 21 societies. *Psychological Science* **29**(9), 1515-1525. (doi:10.1177/0956797618778235).

32. Lima C.F., Anikin A., Monteiro A.C., Scott S.K., Castro S.L. 2019 Automaticity in the Recognition of Nonverbal Emotional Vocalizations. *Emotion* **19**, 219-233.

33. Sauter D.A., Eisner F., Ekman P., Scott S.K. 2010 Cross-cultural recognition of basic emotions through nonverbal emotional vocalizations. *PNAS* **107**, 2408–2412.
